# Reconceptualizing glioblastoma immunotherapy: a four-pillar framework to overcome multidimensional resistance

**DOI:** 10.3389/fmed.2026.1848364

**Published:** 2026-06-17

**Authors:** Naibijiang Abuliezi, Jiale Zhao, Yihan Sun, Tayier Tuersong, Xiaoyi Dun, Qinfen Wu

**Affiliations:** 1 The Second Affiliated Hospital of Xinjiang Medical University, Urumqi, China; 2 Department of Pharmacy, The Second Affiliated Hospital of Xinjiang Medical University, Urumqi, Xinjiang, China; 3 The Fifth Affiliated Hospital of Xinjiang Medical University, Urumqi, China; 4 Xinjiang Key Laboratory of Neurological Disorder Research (Lab No.: XJDX1711), Urumqi, China; 5 Xinjiang Clinical Research Center for Neurological Diseases, Urumqi, China

**Keywords:** glioblastoma, immune checkpoint inhibitors, immune evasion, tumor microenvironment, tumor vaccines

## Abstract

Glioblastoma (GBM) remains a recalcitrant primary brain malignancy characterized by a profound immunosuppressive tumor microenvironment (TME), low mutational burden, and extreme molecular heterogeneity. Despite transformative successes in other solid tumors, clinical efficacy in GBM remains modest due to multifaceted barriers, including impaired antigen presentation, dominant myeloid suppression, and the restrictive blood–brain barrier (BBB). This review provides a comprehensive synthesis of the immunotherapeutic landscape, encompassing checkpoint blockade, vaccines, oncolytic virotherapy, and adoptive cellular therapies. Diverging from traditional modality-centric descriptions, we propose an integrated framework organized around four core pillars of antitumor immunity: enhancing antigen presentation, reversing T cell dysfunction, reprogramming the suppressive TME, and engineering effective intracranial delivery. We detail mechanistic rationales and prioritize recent clinical breakthroughs, including bispecific T-cell engagers and the 2024–2025 milestones in bivalent and TEAM-CAR T cell designs. By delineating mechanisms of resistance and identifying areas of therapeutic convergence, we aim to outline research priorities to render the “cold” GBM niche permissive to durable antitumor immunity.

## Highlights

- GBM has a unique and complex immune-resistant microenvironment and biological characteristics, posing core challenges for immunotherapy.
- Current GBM immunotherapy strategies have adopted a multi-platform approach, with both progress and limitations.
- Combination immunotherapy, along with precision and engineered innovations, is a key direction to overcome the treatment bottleneck in GBM.

## 1 Introduction

Glioblastoma (GBM) is the most common and aggressive primary malignant brain tumor in adults, representing about 14.5% of all central nervous system (CNS) tumors and nearly half of all malignant gliomas (1). Histologically, gliomas are categorized into low-grade (WHO grade 1-2) and high-grade (WHO grade 3-4) subtypes (2, 3). Classified as a grade 4 IDH-wildtype diffuse glioma, GBM is characterized by rapid progression, invasiveness, and extensive molecular heterogeneity. Despite advances in neuroimaging, surgical resection, and adjuvant therapies, GBM remains the archetypal high-grade glioma carrying a dismal prognosis and a median overall survival of approximately 14–16 months with standard-of-care treatment (maximal safe resection, radiotherapy, and temozolomide), though outcomes are highly variable by O^6^-methylguanine-DNA methyltransferase (MGMT) promoter methylation status and molecular subtype (4, 5). In the U.S., GBM has an incidence of 3.23 per 100,000 individuals, with over 12,000 new cases annually (1, 6). While the disease predominantly affects the elderly (55% of cases occur in individuals over 65), younger-onset cases (typically defined as <45 years) are less common but associated with better survival outcomes. Under the 2021 WHO classification, this demographic frequently encompasses IDH-mutant grade 4 astrocytomas, which contribute to the improved prognosis compared to IDH-wildtype GBM (7).

GBM’s resistance to treatment is driven by both intrinsic and acquired genomic alterations, such as telomerase reverse transcriptase (TERT) promoter mutations and chromosomal abnormalities (+7/−10) in late-onset cases, correlating with poorer prognosis (8). In contrast, younger patients—particularly those with IDH-mutant grade 4 astrocytomas—more often harbor ALT-associated mutations such as ATRX and TP53, correlating with more favorable responses to therapy (9). Despite the standard of care established by the Stupp protocol—comprising maximal safe resection, radiotherapy, and temozolomide (TMZ)—treatment efficacy remains limited, with relapse being almost inevitable. While MGMT promoter methylation status serves as a critical predictor of TMZ sensitivity, mechanisms such as mismatch repair (MMR) deficiency, epigenetic plasticity, and acquired alterations in DNA repair pathways drive the development of therapeutic resistance. This challenge is further compounded by the blood–brain barrier (BBB) and an immunosuppressive TME, which together impede effective drug delivery and immune-based interventions (10, 11).

Immunotherapy has reshaped oncology in selected solid tumors—including melanoma, non**-**small cell lung cancer, renal cell carcinoma, and Hodgkin lymphoma—where immune checkpoint inhibitors (ICIs), CAR T cells, vaccines, and oncolytic virotherapy have produced durable responses and, in some cases, cures (12, 13), thereby providing a strong rationale for exploration in GBM (14). Yet GBM presents distinct obstacles that help explain the variable—and often modest—activity observed to date: (1) impaired immune recognition, with low tumor mutational burden and sparse neoantigens limiting T cell priming; GSCs further downregulate antigen-presentation machinery and secrete suppressive mediators (15–18); (2) an immunosuppressive TME enriched for glioma-associated microglia/macrophages (GAMs), myeloid-derived suppressor cells (MDSCs), and regulatory T cells, within a central nervous system that actively regulates immunity and sustains immune-excluded niches (19, 20); and (3) delivery constraints, as the blood–brain barrier and abnormal tumor vasculature impede trafficking and distribution of immune effectors and biologics (17). These features frame the rationale for current hypothesis-driven combinations and delivery strategies evaluated in this review.

In addition to microenvironmental constraints, intrinsic resistance programs—including mismatch repair (MMR) deficiency, p53 pathway dysregulation, and impaired DNA damage-response signaling—further contribute to immune escape. For instance, MMR deficiency leads to microsatellite instability (MSI) and elevated mutational load in a subset of GBMs. Additionally, aberrant DNA damage response can upregulate PD-L1 and suppress interferon signaling, thereby impairing T cell priming (21, 22). Since 2016, several modalities have produced early preclinical or clinical signals, including oncolytic virotherapy (e.g., DNX-2401), CAR T cells directed at EGFRvIII, IL13Rα2, or HER2, and personalized neoantigen vaccines (21, 23, 24). Building on these observations, current work increasingly emphasizes rational combinations that aim to enhance antigen presentation, mitigate T cell exhaustion, reprogram the suppressive TME, and improve delivery across the blood–brain barrier. Collectively, these efforts mark a shift from single agents toward mechanistically informed, multimodal strategies tailored to GBM’s molecular and immunologic heterogeneity, while recognizing that durable clinical benefit remains to be demonstrated.

GBM remains stubbornly lethal; immune evasion within the blood–brain barrier and a suppressive TME limit therapeutic efficacy. This review synthesizes current lines of investigation in GBM immunotherapy—checkpoint blockade, CAR T cells, oncolytic virotherapy, and personalized neoantigen vaccination—alongside emerging combination and biomarker frameworks. Our goal is to delineate mechanisms, areas of convergence, and testable hypotheses that may help render “cold” GBM niches more permissive to immune attack.

## 2 Characteristics of the glioma TME and immunotherapy resistance

**Figure 1:**
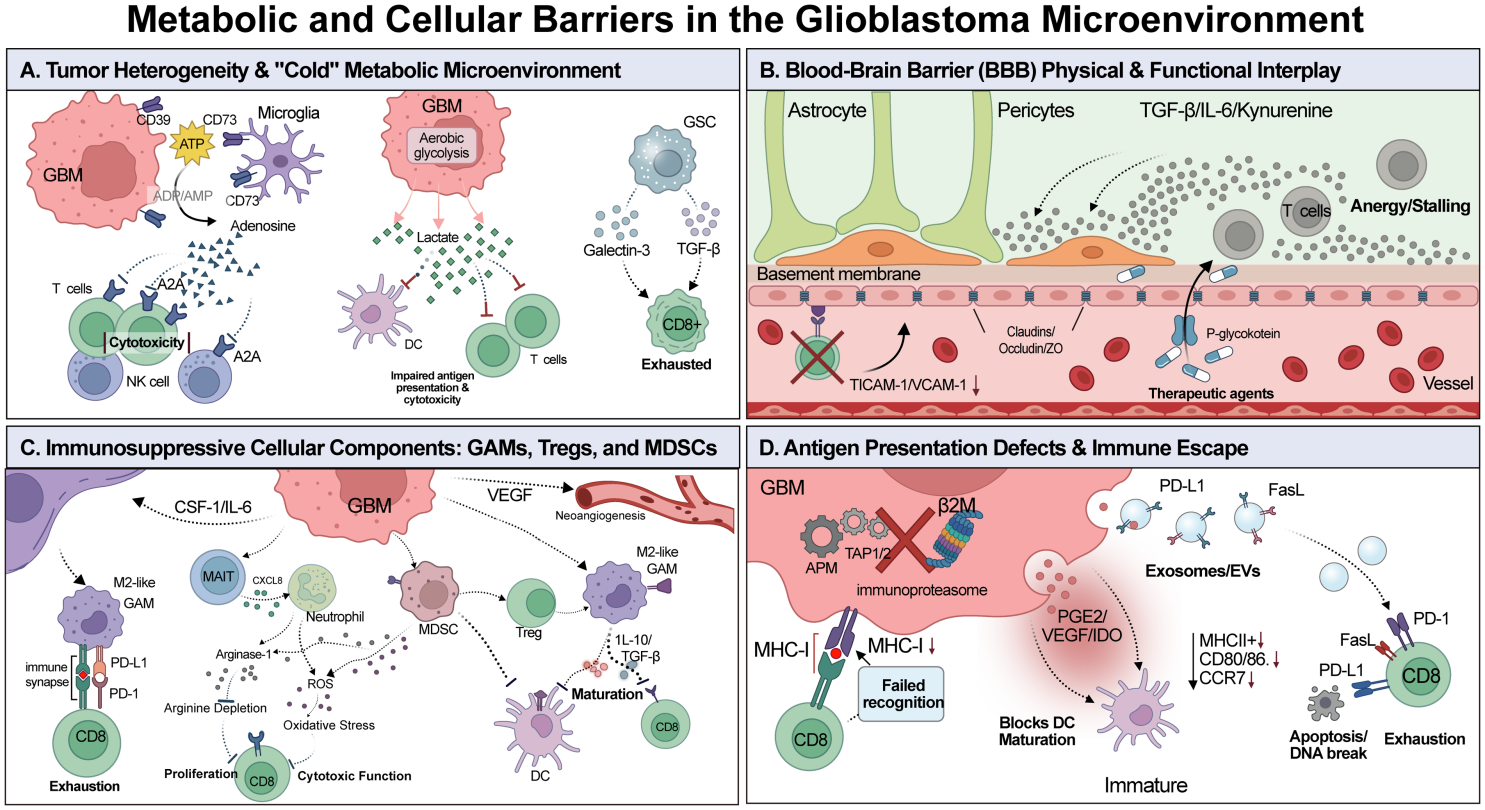
Metabolic, cellular, and immunological barriers driving immune suppression and therapeutic resistance in the GBM microenvironment. **(A)** Tumor heterogeneity and the “cold” metabolic microenvironment. GBM and GSCs utilize aerobic glycolysis to release lactate and acidify the extracellular space. The CD39/CD73 axis catabolizes extracellular ATP into adenosine, which binds A_2A_ receptors to blunt T cell/NK cell cytotoxicity and disrupt DC antigen presentation. Factors like galectin-3 and TGF-β further maintain this immunologically silent state. **(B)** Blood–brain barrier (BBB): physical and functional immune restriction. The neurovascular unit, comprising endothelial cells stabilized by tight junctions (occludin, claudins, ZO-1), restricts the entry of therapeutics and leukocytes. Patchy permeability—intact at infiltrative margins but leaky in the core—creates spatial sanctuary sites. Low expression of ICAM-1/VCAM-1 and astrocyte-derived suppressive factors (IL-6, kynurenine) promote T cell anergy and perivascular stalling. **(C)** Immunosuppressive cellular components: GAMs, Tregs, and MDSCs. Tumor-secreted cues (CSF-1, periostin) polarize GAMs toward M2-like phenotypes and trigger MMP9-mediated neoangiogenesis. These cells, alongside MDSCs and Tregs, release IL-10 and TGF-β. Notably, MAIT cell-derived CXCL8 recruits neutrophils, which release ROS and arginase-1 to deplete arginine, directly inhibiting CD8^+^T cell proliferation. **(D)** Antigen presentation defects and immune escape. GBM cells downregulate the antigen-processing machinery (TAP1/2, β2M) and MHC-I/II, evading adaptive recognition. Infiltrating DCs remain in an immature state (CD80/86 ^low^), failing to prime effective T cell responses. Tumor-derived extracellular vesicles (EVs) further propagate suppression by delivering PD-L1, FasL, and IDO to induce systemic T cell apoptosis and exhaustion.

GBM is among the most treatment-refractory solid tumors. A central determinant is an immunosuppressive, spatially—and temporally—heterogeneous TME that restricts priming, trafficking, and effector function (25, 26). Rather than merely immune-excluded, GBM niches actively enforce tolerance through myeloid-dominant suppression, T cell dysfunction, impaired antigen presentation, hostile metabolic cues, and abnormal vasculature, collectively blunting responses to conventional therapies and immunotherapies (27). Clarifying these features and their interactions provides the framework for hypothesis-driven combinations and delivery strategies discussed below (Figure 1).

### 2.1 Tumor heterogeneity, low immunogenicity, and “cold tumor” phenotype

GBM is one of the most genetically, epigenetically, and transcriptionally heterogeneous tumors (28). The prevailing pattern is non-inflamed (“cold”): sparse T cell infiltration, low MHC class I, limited neoantigen burden, and myeloid-skewed composition. Unlike “hot” tumors such as melanoma, GBM rarely supports spontaneous immune priming. Consequently, single-agent checkpoint blockade has shown only modest activity, as local inertia often prevents durable effector control (27, 29).

The antigenic landscape of GBM further constrains immune recognition. GBM has one of the lowest tumor mutational burdens (TMB) among solid tumors (~1–2 mutations/Mb), yielding few high-quality neoantigens capable of priming CD8⁺ T cells responses. Crucially, this scarcity of targets is compounded by the active downregulation of essential immune components, such as MHC class I molecules and antigen-processing machinery (APM), which renders even existing antigens invisible to the immune system. While shared tumor-associated antigens (TAAs)—including EGFRvIII and IL13Rα2—are present, their highly mosaic distribution allows for subclonal “antigenic escape.” Glioblastoma stem cells (GSCs) further reinforce this evasion. By occupying perivascular sanctuaries and secreting mediators such as galectin-3, periostin, and TGF-*β*, GSCs actively suppress local immunity and maintain a reservoir of therapy-resistant cells (30, 31).

Tumor metabolism enforces this state of immune quiescence, particularly through the CD39/CD73/adenosine axis. This pathway functions as a critical metabolic crosstalk between GBM cells and tumor-associated microglia. During cellular stress or death, GBM cells release extracellular ATP (eATP), which typically acts as a pro-inflammatory “danger signal.” However, this signal is rapidly subverted: CD39 and CD73—co-expressed by both tumor cells and infiltrating microglia—work in tandem to hydrolyze eATP into immunosuppressive adenosine. The resulting high extracellular adenosine levels signal through A_2A_ receptors on T cells and NK cells suppressing effector cytokine production, promoting Treg differentiation, and deepening exhaustion and tolerance (32, 33).

Spatial and temporal heterogeneity further compound these effects. Single-cell studies reveal coexisting lymphocyte-excluded regions, exhausted T-cell zones, and tolerogenic dendritic-cell niches within the same tumor (34). This architectural complexity, paired with the temporal evolution of the tumor under treatment pressure, complicates uniform targeting and fosters resistant outgrowths.

These interacting mechanisms collectively define the “cold” GBM phenotype. Converting this environment into one that is permissive to T cells requires a multi-pronged mechanistic approach. First, the neoantigen repertoire must be expanded; for instance, epigenetic modulators can de-repress silenced tumor antigens or endogenous retroviruses to increase visibility. Second, metabolic barriers must be dismantled, such as by inhibiting the adenosine axis to restore the metabolic fitness of infiltrating CD8 + T cells and NK cells. Finally, innate immunity must be “re-primed” through danger signals like STING agonists to overcome local inertia and recruit fresh effectors into the tumor (35). These strategies are discussed in detail within the context of specific therapeutic applications in Sections 3 and 5.3.

### 2.2 Immunosuppressive cellular components: GAMs, Tregs, and MDSCs

GBM features abundant immunosuppressive myeloid and regulatory lymphoid cells that blunt priming, infiltration, and effector function locally and systemically, reinforcing tolerance and therapy resistance (36–42).

*GAMs:* Glioma-derived cues (e.g., CSF-1, IL-6, periostin) skew GAMs toward M2-like programs that upregulate PD-L1 and secrete immunoregulatory mediators (e.g., IL-10, TGF-β, prostaglandin E2), suppressing cytotoxic T cells and dendritic-cell maturation (36, 37). Beyond cytokine-mediated suppression, GAMs facilitate tumor invasion through matrix metalloproteinase-9 (MMP9) and drive pathological neoangiogenesis via VEGF. This dysfunctional vasculature paradoxically impairs lymphocyte trafficking while supporting tumor growth (38). This myeloid-driven suppression is further amplified by unconventional T-cell subsets, such as mucosal-associated invariant T (MAIT) cells. In the GBM TME, MAIT cells secrete CXCL8 to recruit neutrophils, which in turn release reactive oxygen species (ROS) and arginase-1. This axis depletes essential arginine and generates oxidative stress, effectively paralyzing effector T-cell proliferation (39–42).

*Regulatory T cells (Tregs)*: Tregs are enriched in GBM and cooperate with GAMs to enforce tolerance (43–46). Their suppressive toolkit includes contact-dependent inhibition via high CTLA-4 expression, activation of the adenosinergic *CD*39/*CD*73 pathway, and the release of IL-10 and TGF-β that dampens T-cell receptor signaling. GSCs further sustain this niche by secreting specific chemoattractants that preferentially recruit and maintain Tregs within the perivascular space (21). Clinically, greater Treg infiltration associates with worse outcomes and limited checkpoint responsiveness (43–46).

*MDSCs*: Recruited by CCL2, CXCL8, and GM-CSF, MDSCs inhibit T-cell proliferation and function through arginase-1, ROS, and nitric oxide (iNOS) and impair dendritic-cell antigen presentation (47–50). They also promote Treg differentiation and bias macrophages toward suppressive states, amplifying network effects (51). Elevated circulating CD33⁺HLA-DR^low^ MDSCs correlate with progression, reduced T-cell infiltration (41). In preclinical models, depleting or reprogramming MDSCs—e.g., with ATRA or CSF-1R pathway modulation—restores elements of antitumor immunity and enhances ICI or vaccine activity. Clinically, the CSF-1R inhibitor pexidartinib (PLX3397) was evaluated in a Phase II study in recurrent GBM (NCT01349049), demonstrating tumor-infiltrating macrophage reduction and acceptable tolerability; however, single-agent efficacy was modest, underscoring that CSF-1R blockade will likely require combination with T cell-activating strategies to achieve durable antitumor immune responses (50).

*Summary of Network Effects*: GAMs, Tregs, and MDSCs form a mutually reinforcing barrier to effective immunity (52). Targeting recruitment/polarization (CSF-1/CSF-1R), metabolic checkpoints (CD39/CD73–A_2A_), effector-suppressive enzymes (arginase-1/iNOS), and neutrophil trafficking are testable strategies; given context-specific roles and compensations, biomarker-guided, temporally staged combinations will likely be required for durable modulation.

### 2.3 Blood–brain barrier (BBB): a physical and functional obstacle

The BBB is a selective neurovascular interface that restricts entry of antibodies, cells, and many small molecules, thereby limiting the impact of systemically delivered immunotherapies (53). In GBM, permeability is patchy: angiogenic cores may be leaky, whereas infiltrative margins often retain an intact barrier. This spatial heterogeneity yields uneven drug and cell distribution, sanctuary regions for tumor cells, and dosing uncertainty that collectively blunt responses to ICIs, CAR T cells, and monoclonal antibodies (54, 55).

Beyond passive exclusion, the BBB actively regulates immunity. Tight junctions (claudins, occludin, ZO proteins) restrict paracellular flux; low endothelial ICAM-1/VCAM-1 diminishes leukocyte adhesion and diapedesis; and efflux transporters (e.g., P-glycoprotein and other ABC family members) lower intracranial drug exposure. Astrocytes and pericytes further reinforce suppression through TGF-β and IL-6 signaling (56). Even activated lymphocytes that traverse the barrier may stall in perivascular spaces enriched for inhibitory cytokines and metabolites (adenosine, kynurenine), promoting anergy or exhaustion; GBM and associated glia can also upregulate PD-L1 in response to IFN-*γ*, attenuating cytotoxic function via checkpoint engagement (57, 58).

Accordingly, delivery-focused strategies are under active study: transient BBB opening with focused ultrasound and microbubbles; convection-enhanced or intraparenchymal delivery; and nanoparticle platforms that leverage receptor-mediated transcytosis (59). Cellular and vascular approaches—including chemokine-receptor engineering of T cells and attempts to reprogram the endothelial–astrocyte axis—aim to facilitate trafficking and intratumoral persistence (60). While evidence remains preliminary, treating the BBB as a modifiable interface offers testable paths to improve intracranial exposure and, potentially, the effectiveness of immunotherapy in GBM.

### 2.4 Antigen presentation deficiency and immune escape

Effective anti-tumor immunity depends on robust antigen presentation to initiate and sustain cytotoxic T cell responses. In GBM, several mechanisms subvert this process. Tumor cells frequently downregulate key components of the antigen-processing machinery (APM), including TAP1/2, β2-microglobulin (β2M), and immunoproteasome subunits (61). These deficiencies hinder the loading of tumor-derived peptides onto MHC class I molecules, diminishing their surface expression and recognition by CD8⁺ T cells. Loss or mutation of β2M, commonly observed in GBM, correlates with immune checkpoint blockade resistance and immune escape (62).

Antigen-presenting cells (APCs), particularly dendritic cells (DCs), within the glioma microenvironment are also functionally impaired. Glioma-infiltrating DCs often display an immature phenotype, marked by low MHC II, CD80/CD86, and CCR7 expression, which impairs both T cell priming and migration to lymph nodes (63). Immunosuppressive cytokines (IL-10, TGF-β) further skew DCs toward a tolerogenic state. Tumor-derived factors like PGE2, VEGF, and IDO inhibit DC maturation, promoting T cell anergy or the differentiation of regulatory T cells (Tregs).

Epigenetic alterations play a central role in immune evasion, as mutations in interferon regulatory elements or SOCS family genes impair type I and II IFN signaling, undermining MHC upregulation and antigen cross-presentation (64). These defects reduce GBM and APC responsiveness to immunostimulatory cues, further evading adaptive immunity (41). Moreover, extracellular vesicles (EVs) secreted by GBM cells contribute to immune suppression. These vesicles, carrying PD-L1, FasL, and TGF-*β*, interact with immune cells at distant sites, inducing T cell exhaustion (via PD-L1), triggering T cell apoptosis (via FasL), and promoting Treg differentiation and NK cell dysfunction (via TGF-β) (65).

Targeting these immune evasion mechanisms presents an opportunity for therapeutic intervention. Approaches such as epigenetic modulation (e.g., DNMT or HDAC inhibitors) to reactivate silenced antigens, STING/TLR agonists to boost APC function, and engineered T cell therapies or vaccines that bypass conventional MHC presentation could restore effective anti-tumor responses (66). While antigen presentation deficiencies remain a significant hurdle, these vulnerabilities could be leveraged to enhance therapeutic efficacy and overcome immune escape in GBM.

## 3 Immunotherapeutic strategies for GBM: current landscape and future directions

GBM’s TME upregulates canonical and emerging checkpoints (PD-1/PD-L1, CTLA-4, LAG-3, TIM-3), yet multiple phase III ICI trials failed to improve survival, underscoring intrinsic immune resistance (67–73).

**Figure 2:**
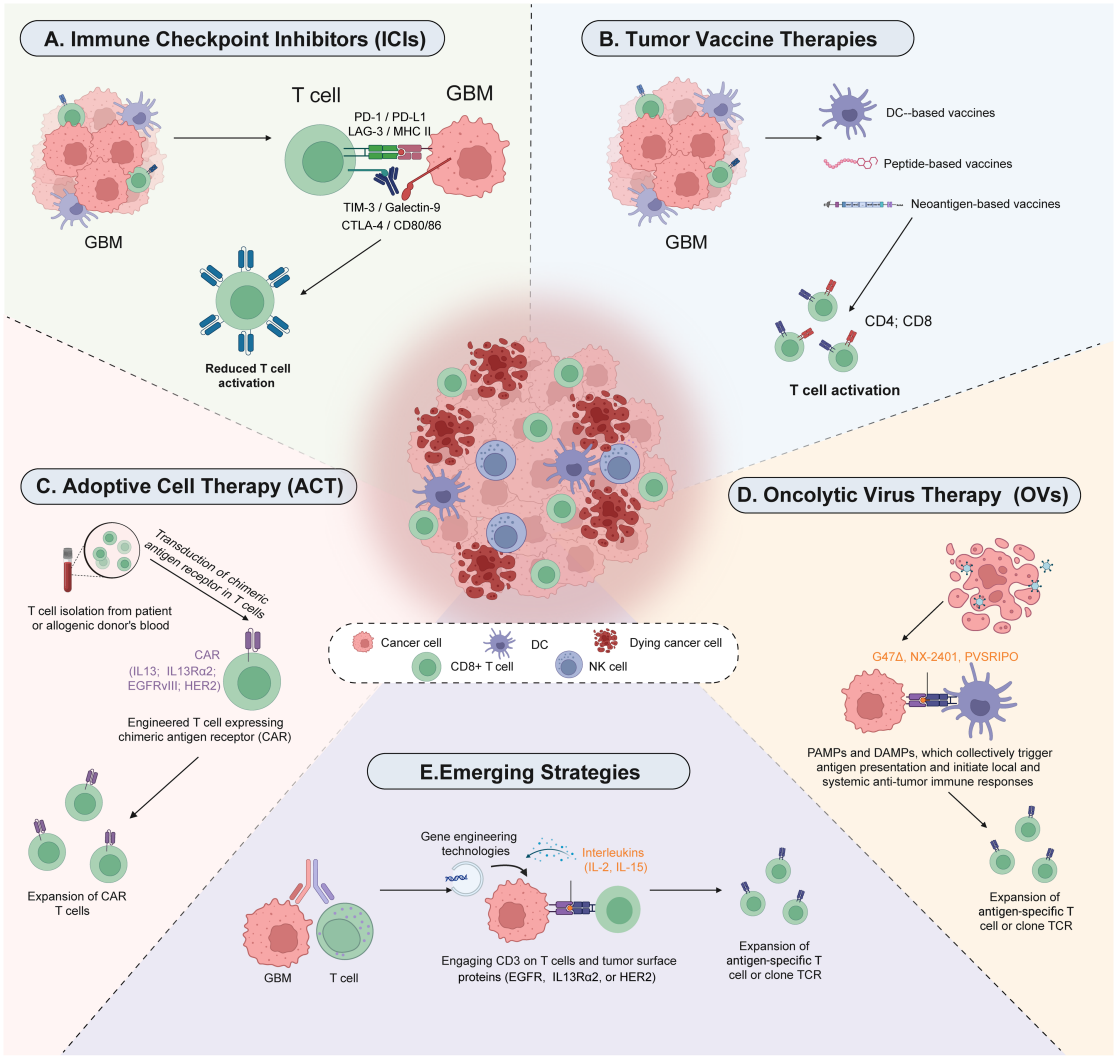
Major immunotherapeutic strategies and their target barriers in GBM. **(A)** Tumor heterogeneity, low immunogenicity, and ‘cold tumor’ phenotype—depicting the metabolic (aerobic glycolysis, lactate, CD73/CD39-adenosine axis) and antigenic (MHC I downregulation, necrosis-associated DAMPs) mechanisms that contribute to immune quiescence, illustrating the reciprocal relationships between GBM cells and effector T/NK cells. **(B)** Tumor Vaccine Therapies Leverage GBM-derived antigens to activate T cells via three common platforms: dendritic cell (DC)-based vaccines, peptide-based vaccines, and neoantigen-based vaccines, driving antigen-specific CD4⁺/CD8⁺ T cell responses. **(C)** Adoptive Cell Therapy (ACT) Involves isolating T cells from patients (or allogeneic donors), engineering them to express chimeric antigen receptors (CARs) targeting GBM-associated antigens (e.g., IL13Rα2, EGFRvIII, HER2), expanding the CAR-T cells ex vivo, and infusing them back to target GBM. **(D)** Oncolytic Virus Therapy (OVs)Utilizes engineered viruses (e.g., G47Δ, DNX-2401, PVS-RIPO) that selectively infect and lyse GBM cells. This process releases pathogen-associated molecular patterns (PAMPs) and damage-associated molecular patterns (DAMPs), triggering innate and adaptive anti-tumor immunity and expanding antigen-specific T cells (e.g., TCR T cells). **(E)** Emerging StrategiesCombine gene engineering technologies (e.g., for cytokine delivery, e.g., IL2/IL-15) and bispecific agents that engage CD3 on T cells with GBM surface proteins (e.g., EGFR, IL13Rα2, HER2), promoting localized T cell expansion and anti-GBM activity.

Accordingly, the field is pivoting to biomarker-guided combinations—ICIs with radiotherapy/anti-angiogenics/vaccines, adoptive cell therapies, oncolytic viruses, and emerging modalities (bispecifics, localized cytokines, metabolic/TME reprogramming)—to convert “cold” GBM into a responsive state (74–121) (Figure 2).

### 3.1 Immune checkpoint inhibitors (ICIs)

GBM’s immunosuppressive TME upregulates various immune checkpoint pathways, including PD-1/PD-L1 and CTLA-4, which impair T cell activation and function through interactions between tumor and immune cells (66–69). Emerging checkpoints like LAG-3 and TIM-3, often co-expressed with PD-1, further inhibit cytotoxic T lymphocyte (CTL) activity, and may represent high-priority targets for therapeutic intervention (70). Despite the success of immune checkpoint inhibitors (ICIs) in other malignancies, their application in GBM has been limited, with pivotal trials demonstrating no overall survival (OS) benefit in unselected populations. CheckMate 143 (NCT02017717, n = 369) compared nivolumab versus bevacizumab in recurrent GBM; the median OS was 9.8 versus 10.0 months (HR = 1.04, 95% CI: 0.83–1.30), with no survival advantage and higher toxicity in the nivolumab arm, establishing that single-agent PD-1 blockade is insufficient in unselected recurrent GBM (74). In the newly diagnosed setting, CheckMate 498 (NCT02617589, n = 560) evaluated nivolumab plus RT versus temozolomide (TMZ) plus RT in MGMT-unmethylated tumors. Median OS was 13.4 versus 14.9 months (HR = 1.31, *p* = 0.0037), with nivolumab proving inferior to TMZ despite the *a priori* hypothesis that MGMT-unmethylated tumors might represent a more ICI-responsive population. Similarly, the CheckMate 548 trial (NCT02667587, *n* = 716) investigated the addition of nivolumab to standard TMZ and RT in MGMT-methylated GBM, reporting a median progression-free survival (PFS) of 10.6 versus 10.3 months (HR = 1.00, 95% CI: 0.70–1.04, *p* = 0.11), ultimately failing to demonstrate clinical benefit. These consistent failures across recurrent and newly diagnosed settings, irrespective of MGMT methylation status, underscore that GBM’s immunosuppressive TME and low neoantigen burden create profound primary resistance to checkpoint monotherapy (71–73).

Nonetheless, ICIs remain an active area of investigation in GBM immunotherapy, with ongoing efforts to identify patient subsets and combination contexts in which meaningful clinical benefit may be achievable, despite the lack of demonstrated survival benefit in unselected populations to date. These therapies are therefore being integrated into mechanistically informed combination strategies: (1) Radiotherapy (RT) + ICI: RT induces immunogenic tumor cell death, upregulates MHC class I and PD-L1 on surviving tumor cells, and releases tumor antigens that can prime or restimulate T cells—creating a rationale for sequential checkpoint blockade to prevent the adaptive PD-L1 resistance induced by RT. However, the Phase III CheckMate 498 trial demonstrated that even in the most RT-centric context (newly diagnosed GBM, RT + nivolumab without TMZ), ICI did not improve OS vs. standard chemoradiotherapy (OS 13.4 vs. 14.9 months, HR = 1.31), suggesting that additional TME-targeting strategies may be needed to unlock RT–ICI synergy. (2) Anti-angiogenic normalization (bevacizumab) + ICI: VEGF blockade reduces intratumoral hypoxia and interstitial pressure, upregulates endothelial ICAM-1/VCAM-1, and improves lymphocyte adhesion and diapedesis into the tumor—effects potentially additive with ICI-mediated T cell reactivation. Despite this rationale, CheckMate 143 showed nivolumab + bevacizumab was not superior to bevacizumab alone in recurrent GBM (OS 10.5 vs. 10.0 months, HR = 1.08). The bevacizumab combination remains of interest specifically for its immunological rather than anti-tumor role in selected populations. (3) Neoantigen-based vaccines + ICI: Vaccines prime antigen-specific T cells while ICI counteracts the checkpoint-mediated suppression that would otherwise dampen vaccine-induced T cell responses; this combination has shown immunological activity (neoantigen-specific T cell expansion + IFN-*γ* production) in early GBM studies, with prospective evaluation ongoing in NCT04280848 (74–76). Furthermore, ICIs are being combined with engineered cellular therapies such as CAR T and TCR T cells. These modified immune cells, though highly effective in tumor targeting, face the same suppressive forces as conventional T cells. ICIs can counteract these barriers by enhancing CAR T cell persistence and cytotoxic activity in GBM’s hostile environment. Emerging checkpoint targets beyond PD-1/CTLA-4 include LAG-3, TIM-3, and TIGIT, which are co-expressed on exhausted intratumoral T cells and represent high-priority targets for combinatorial blockade. Notably, CD47—the “don’t eat me” signal that inhibits macrophage-mediated phagocytosis—represents a mechanistically distinct, non-T cell checkpoint; anti-CD47 strategies function by relieving phagocytic suppression on myeloid cells rather than reinvigorating T cells. With respect to predictive biomarkers for ICI benefit in GBM: MMR deficiency (dMMR)/MSI-high status is clinically actionable (eligible for pembrolizumab under FDA tumor-agnostic approval based on KEYNOTE-158); PTEN loss and MAPK pathway alterations associate with immunosuppressive TME phenotypes; and TME immunophenotyping into myeloid-enriched vs. lymphocyte-inflamed subtypes may serve as a patient stratification framework for future ICI trials. With biomarker-guided combination strategies, these therapies hold potential for overcoming GBM’s multifaceted resistance mechanisms, though large randomized, biomarker-stratified clinical trials remain critical to define how ICIs can best overcome the T-cell Dysfunction pillar within the broader immunosuppressive TME (77–79).

### 3.2 Tumor vaccine therapies

Tumor vaccines represent a mechanistically diverse class of immunotherapies designed to prime or amplify antigen-specific T cell responses against GBM. They are categorized into three principal platforms: (1) dendritic cell (DC)-based vaccines, which involve ex vivo antigen loading of patient-derived DCs followed by reinfusion to prime T cell responses; (2) peptide-based vaccines, targeting either shared tumor-associated antigens (TAAs) or patient-specific mutation-derived neoantigens; and (3) mRNA-based vaccines, a rapidly deployable platform that encodes tumor antigens directly in lipid nanoparticle-formulated mRNA for endogenous DC uptake and cross-presentation. Each platform has distinct mechanistic profiles, manufacturing requirements, and clinical evidence bases, as outlined below (80). DC-based vaccine clinical trials have generated important, if mixed, results. ICT-107, a DC vaccine targeting six GBM-associated antigens (HER2/neu, TRP-2, gp100, AIM2, MAGE-1, IL13Rα2, AIM-2), showed a statistically significant improvement in progression-free survival in HLA-A2-positive patients in Phase II (NCT01280552: PFS 11.2 vs. 9.0 months, *p* = 0.022), suggesting immunological activity in an antigen-matched population; however, the Phase III trial was terminated due to futility, illustrating the challenge of antigen heterogeneity across unselected GBM populations. DCVax-L (autologous tumor lysate-loaded DC vaccine) was evaluated in a Phase III trial (NCT00045968, n = 331), reporting a median OS of 19.3 months in the intent-to-treat population, with a 3-year OS rate of 30.2% in a subgroup receiving vaccine post-recurrence; methodological considerations regarding the crossover design complicate definitive OS attribution, but the biological signal has generated substantial interest and ongoing investigation. Peptide vaccines, such as rindopepimut (CDX-110) targeting the EGFRvIII neoepitope, failed to show survival benefit in the ACT IV Phase III trial (NCT01480479, *n* = 745). *Post-hoc* analysis revealed EGFRvIII antigen loss in 82% of post-treatment recurrent tumors, directly demonstrating antigen escape as the primary failure mechanism and underscoring the need for multi-antigen vaccine strategies (81, 82).

mRNA vaccines represent a mechanistically compelling platform for GBM: lipid nanoparticle-encapsulated mRNA is taken up by antigen-presenting cells (particularly dendritic cells), translated into tumor-specific proteins, and processed via both MHC class I (for CD8⁺ T cell priming) and MHC class II (for CD4⁺ T helper activation) pathways. Compared to peptide vaccines, mRNA vaccines have the advantage of encoding long antigens that allow endogenous antigen processing, can be designed to include both MHC-I and MHC-II epitopes, and can be manufactured rapidly from sequencing data. Limitations include mRNA instability, variable transfection efficiency in immunosuppressed GBM patients, and the need for adjuvant co-delivery to achieve sufficient DC activation. In GBM, mRNA vaccines are being explored as neoantigen platforms (87). These vaccines aim to activate both CD4⁺ and CD8⁺ T cell responses and promote immunological memory. Tumor-associated antigens (TAAs) like EGFRvIII and WT1 have been extensively studied, but challenges such as antigen loss and heterogeneity limit their long-term efficacy (91, 92). Neoantigen vaccines, based on unique tumor mutations, offer an alternative by targeting more stable and individualized epitopes. Early trials of neoantigen vaccines have shown immunogenicity, with T cell expansion targeting multiple epitopes, though practical challenges such as production time, cost, and epitope coverage persist (87).

Neoantigen vaccines target tumor-specific mutant peptides with limited central tolerance. Landmark Phase I studies (122, 123) demonstrated personalized neoantigen vaccines generating vaccine-reactive CD4^+^ and CD8^+^ T cells with intratumoral trafficking capacity in newly diagnosed GBM patients. Key challenges include: (a) epitope coverage — each GBM harbors ~30–50 candidate neoantigens but only ~20–30% elicit immunogenic T cell responses; (b) production timelines of 3–8 weeks from tumor sequencing to vaccine manufacture; and (c) clonal vs. subclonal neoantigen prioritization — targeting clonal mutations (present in all tumor cells) maximizes the probability of universal tumor-cell coverage. Neoantigen vaccine + anti-PD-1 combinations are under active evaluation (e.g., NCT04717401) leveraging AI-based neoantigen prioritization pipelines (NetMHCpan, pVACtools).

Recent advances emphasize the importance of CD4⁺ T helper cells, which are crucial for sustaining and amplifying anti-tumor immunity. CD4⁺ T cells support cytotoxic CD8⁺ T cells, promote DC maturation, and can also exert direct cytotoxic effects within the TME (88, 90). Next-generation vaccines incorporating MHC class II epitopes aim to activate both arms of the immune system for more durable responses. However, tumor heterogeneity and dynamic antigen expression continue to pose significant barriers to lasting efficacy. Two vaccine design principles are particularly important: (1) Antigen heterogeneity and epitope coverage: single-antigen vaccines create selection pressure for antigen-negative escape variants (as demonstrated by EGFRvIII loss post-rindopepimut). Multi-epitope constructs encoding both shared TAAs and patient-specific neoantigens reduce this risk by requiring simultaneous loss of multiple antigens; broader epitope coverage (15–20 neoepitopes per vaccine) is now achievable via AI-driven MHC-binding predictors (e.g., pVACtools, NetMHCpan), though immunodominance hierarchies and antigen editing remain active monitoring priorities in ongoing trials (e.g., NCT04280848). (2) Adjuvant selection: poly-ICLC (a TLR3/MDA5 agonist promoting DC maturation and Th1 skewing) and GM-CSF are the most widely used adjuvants in GBM vaccine trials; their optimal dosing, scheduling, and combination with vaccines requires further study, as over-adjuvantation can paradoxically induce regulatory responses. Epitope spreading—the induction of *de novo* T cell responses against non-targeted tumor antigens following vaccine-induced tumor cell death—has been documented post-DCVax-L and represents a secondary immune-broadening mechanism that may contribute to durable responses in a subset of patients. Strategies to overcome these challenges include multi-epitope vaccines, epitope spreading, and combination therapies with checkpoint inhibitors or adjuvants.

### 3.3 Adoptive cell therapy (ACT)

Adoptive cell therapy (ACT), particularly CAR T and TCR T cell therapies, offers a promising approach for GBM treatment by genetically modifying T cells to target tumor-specific antigens. CAR T cells utilize synthetic receptors to recognize tumor-associated surface antigens, facilitating MHC-independent tumor recognition (93). In GBM, targets such as IL13 receptor α2 (IL13Rα2), EGFRvIII, and HER2 have defined the current landscape. For instance, IL13Rα2-directed CAR T cells (NCT02208362) achieved a complete radiographic response lasting 7.5 months in a landmark case study involving intraventricular and intratumoral infusions; however, subsequent antigen-loss escape in this patient motivated the shift toward bivalent targeting. Similarly, EGFRvIII-directed CAR T cells (NCT01454596) demonstrated successful intracranial accumulation, but their efficacy was hindered by rapid post-treatment EGFRvIII downregulation across multiple cohorts. Furthermore, HER2-directed CAR T cells (NCT01109095) showed a median *OS* of 11.1 months in a study of *n* = 17 patients, with stable disease achieved in seven cases, identifying insufficient T cell persistence as a primary limitation. Collectively, these first-generation trials established immunological proof-of-concept while defining antigen loss, insufficient intracranial accumulation, and T cell exhaustion as the three principal barriers to clinical efficacy (94, 95). TCR-engineered T cells (TCR T cells) offer a distinct advantage by targeting intracellular antigens, such as the MAGE-A family or mutant IDH1 R132H, presented on *MHC* molecules. This approach broadens the repertoire of targetable peptides, including neoantigens, although it remains restricted by the patient’s *HLA* type (96, 97). However, the profound heterogeneity of GBM remains a critical hurdle, potentially leading to immune escape when target antigens are lost or downregulated (98). Advances in next-generation CAR T engineering include: (a) armored/TRUCK CARs secreting immune-activating cytokines (IL-12, IL-15) upon antigen engagement; (b) SynNotch/AND-gate logic-gated CARs requiring simultaneous recognition of two antigens for activation, limiting off-tumor toxicity; (c) inhibitory CARs (iCARs) suppressing activation upon normal tissue antigen recognition; and (d) CRISPR/Cas9 disruption of PD-1, LAG-3, or TIM-3 generating exhaustion-resistant CAR T cells entering early-phase solid tumor trials (99).

Emerging alternatives to traditional T cell therapies include CAR-engineered natural killer (CAR NK) cells and CAR-modified macrophages (CAR M). CAR NK cells offer the advantage of recognizing tumor cells in an HLA-independent manner, reducing the risks of graft-versus-host disease (GvHD) and cytokine release syndrome (CRS). In preclinical GBM models, CAR NK cells targeting EGFR, EGFRvIII, IL13Rα2, or GD2 have demonstrated potent, selective cytotoxicity with favorable safety profiles due to their intrinsic short lifespan (89). Crucially, CAR NK cells can mediate natural cytotoxicity against antigen-negative tumor subclones via NKG2D and DNAM-1 activating receptors, providing inherent multi-antigen coverage that single-target CAR T cells lack. While iPSC-derived platforms (e.g., FT596) were initially established in hematologic malignancies, these allogeneic “off-the-shelf” technologies are now being rapidly adapted for GBM (101, 124). Parallelly, CAR-macrophage (CAR M) therapy leverages the innate ability of macrophages to traffic into hypoxic tumor cores and remodel the immunosuppressive myeloid TME. Early clinical evidence from the CT-0508 trial (anti-HER2 CAR M, NCT04660929) has already demonstrated intratumoral persistence in patients with solid tumors.

Recent 2024 clinical breakthroughs have begun to directly address the core challenges of heterogeneity and delivery identified in these platforms. Choi et al. (125) reported a landmark case series of CARv3-TEAM-E cells—a novel construct that simultaneously targets EGFRvIII via a CAR domain and secretes a T-cell-engaging antibody molecule (TEAM) against wild-type EGFR. This tandem-targeting strategy demonstrated rapid radiographic regression in three patients, providing the first clinical proof-of-concept that bispecific intracranial CAR T approaches can overcome the antigen-loss escape that previously limited single-target therapies.

Complementing this work, Bagley et al. (126) reported on a bivalent CAR T construct targeting both EGFR and IL13Rα2, delivered via the intrathecal route. By engaging two of the most validated GBM surface antigens, this strategy addresses the vulnerability of antigen heterogeneity, while the intrathecal route circumvents the blood–brain barrier (BBB) limitations. These studies represent a paradigm shift in GBM therapy, moving from single-target systemic infusion toward multi-antigen, locally delivered approaches that more rationally account for the biological complexity of GBM resistance.

In conclusion, while ACT faces significant hurdles like immune suppression and the TME, the integration of multi-antigen targeting, intrathecal delivery, and novel platforms like CAR NK and CAR M offers a promising roadmap to overcome these barriers and improve patient outcomes.

### 3.4 Oncolytic virus therapy (OVs)

Oncolytic virus (OV) therapy is an innovative approach that combines direct tumor cell lysis with immune activation, offering a dual mechanism to target GBM. Engineered or naturally occurring viruses selectively replicate within tumor cells, sparing normal tissues. As the virus replicates and lyses tumor cells, it simultaneously releases tumor-associated antigens (TAAs), pathogen-associated molecular patterns (PAMPs—viral nucleic acids and proteins recognized by innate immune pattern-recognition receptors such as TLRs and RIG-I), and danger-associated molecular patterns (DAMPs—endogenous molecules released from dying cells, including HMGB1, calreticulin, and ATP, which activate dendritic cells and stimulate adaptive immune priming). This PAMP/DAMP release converts an immunologically “cold” GBM into an inflamed, antigen-rich environment capable of supporting CD8⁺ T cell priming—a mechanism conceptually analogous to immunogenic cell death induced by certain chemotherapies or radiotherapy (103, 112). This ability to transform the immunologically “cold” GBM microenvironment into an immune-reactive state makes OVs a promising therapeutic modality for the disease.

Several viral platforms have been tested in GBM, including herpes simplex virus (HSV), adenovirus, poliovirus, and reovirus (109). Among these, the G47Δ HSV-1 strain, engineered for enhanced tumor selectivity and immune activation, has shown particular promise. Its deletion of ICP47 promotes antigen presentation, enhancing immune visibility. Other promising candidates, such as DNX-2401 (oncolytic adenovirus) and PVSRIPO (live-attenuated poliovirus), have demonstrated encouraging safety and efficacy in early trials (104, 105). A major milestone occurred in 2021 when G47Δ (teserpaturev) received conditional approval in Japan—marking the first oncolytic virus authorized specifically for malignant glioma. This regulatory milestone was supported by Phase II trial data (UMIN000015996) in which G47Δ demonstrated a 1-year survival rate of 84.2% after treatment initiation in patients with residual or recurrent GBM. Furthermore, post-treatment biopsies from these patients provided mechanistic evidence of therapeutic activity through significantly increased tumor-infiltrating lymphocytes. DNX-2401 (tasadenoturev), a conditionally replication-competent oncolytic adenovirus with deletion of the Rb-binding CR2 domain to confer tumor-selectivity, was evaluated in Phase I (NCT00805376) in recurrent GBM: single intratumoral injection produced radiographic regression in 20% of patients, with median OS of 9.5 months and histological evidence of increased TIL infiltration on follow-up biopsy. PVSRIPO (recombinant poliovirus/rhinovirus chimera targeting CD155/PVR expressed on GBM cells) was evaluated in Phase Ib (NCT01491893): the 24-month OS rate of 21% substantially exceeded historical control rates, and the agent received FDA Breakthrough Therapy Designation in 2016 (106).

OV-mediated TME conversion from immunologically cold to immune-reactive occurs through: (a) release of sequestered tumor antigens during lysis; (b) DAMP/PAMP-triggered DC activation and IFN-I signaling; (c) MHC-I upregulation on surviving tumor cells; and (d) adaptive PD-L1 upregulation driven by IFN-gamma, providing mechanistic rationale for OV + ICI combinations. Saha et al. demonstrated synergistic activity of G47Δ and anti-PD-1 in immunocompetent glioma models, exceeding the efficacy of either agent alone (127). OV-induced antigen release can also prime the immune system for neoantigen vaccine strategies, inducing epitope spreading, enhancing T cell diversity, and generating long-term immunological memory (107, 108). Specific ongoing trials evaluating OV combinations in GBM include: NCT02798406 (DNX-2401 + pembrolizumab; Phase II; 52-week OS rate 20.0% with 4 patients alive >15 months); NCT04479241 (PVSRIPO + pembrolizumab; Phase Ib); and NCT02062827 (M032 IL-12-expressing HSV-1 + pembrolizumab; ongoing). These studies highlight the evolving clinical landscape of combining OVs with immune checkpoint inhibitors.

Key delivery strategies being optimized include: (a) convection-enhanced delivery (CED) — neurosurgical positive-pressure catheter technique distributing agents throughout tumor parenchyma by bulk flow, circumventing poor diffusion in large necrotic GBMs; (b) repeated intratumoral dosing to sustain viral replication and antigen release beyond immune clearance of initial infusions; and (c) arming OVs with immune-modifying transgenes — encoding cytokines (IL-12: M032, NCT02062827) or anti-PD-L1 single-chain antibodies into the viral genome enables local immune modulation without systemic toxicity. Future research will focus on patient stratification and the discovery of biomarker, such as baseline TIL density, viral biodistribution markers, to refine these multimodal regimens.

## 4 Emerging and combination immunotherapies

Emerging immunotherapeutic strategies for glioblastoma multiforme (GBM) aim to address key challenges such as immune cell trafficking, persistence, and the immunosuppressive tumor microenvironment (TME). One promising approach is bispecific antibodies (BsAbs), designed to bind tumor antigens and immune cells, redirecting T cells to target tumors directly. BsAbs demonstrate potent anti-tumor activity, even in low-antigen-density environments (110, 111). Localized cytokine delivery systems using nanoparticle encapsulation or antibody fusion constructs target key cytokines including: IL-12 (Th1 polarizer stimulating IFN-gamma and CD8^+^ T cytotoxicity; NCT05100550); IL-15 (promotes NK and memory CD8^+^ T cell expansion without Treg induction; evaluated as fused IL-15/IL-15Ralpha complexes); and IL-2 (T cell growth factor, but dose-limiting systemic toxicity makes localized delivery via antibody fusion or polymer microspheres essential). These selectively expand effector T cells and NK cells within the tumor while minimizing systemic toxicity (114). Reprogramming immune metabolism is another critical focus. Enhancing mitochondrial function and blocking metabolic checkpoints like IDO1 or adenosine signaling can help sustain CAR T or CAR NK cells in the nutrient-deprived, lactate-rich GBM microenvironment (115, 116). Gene-editing strategies to boost immune cell migration and persistence include: (a) CXCR3 or CCR2 overexpression to direct T cell homing toward CXCL10/CCL2 gradients in GBM; (b) CRISPR/Cas9 disruption of TGFBR2 or dominant-negative TGF-beta receptor insertion to confer TGF-beta resistance in the highly immunosuppressive GBM TME; (c) REGNASE-1 knockout to enhance T cell cytokine expression and persistence (128); and (d) PGC-1alpha overexpression to enhance mitochondrial biogenesis and sustain T cell function in the lactate-rich, glucose-depleted GBM metabolic microenvironment.

Glioma-associated macrophages (GAMs), which often have a pro-tumorigenic phenotype, can be reprogrammed using: (a) CSF-1R inhibitors (pexidartinib; see Section 2.2) to shift GAM polarization from M2-like to M1-like; and (b) STAT3 pathway inhibition — STAT3 is a master transcriptional regulator of immunosuppressive myeloid programming; ONC201 (ClpP agonist with STAT3-inhibitory activity; NCT03416530) and WP1066 are under evaluation. These agents address the myeloid suppression that limits CAR T and ICI activity within the GBM TME (118, 119). Tumor vascular normalization represents a critical component of combination therapy: bevacizumab (anti-VEGF) has not improved OS in Phase III trials (AVAGLIO NCT00943826; RTOG0825 NCT00884741), but its mechanistic rationale in combination immunotherapy is distinct from direct anti-tumor cytotoxicity. Normalization of the aberrant GBM vasculature reduces intratumoral hypoxia and interstitial hypertension, upregulates endothelial ICAM-1/VCAM-1 expression, and improves lymphocyte adhesion and diapedesis into the tumor—effects that may be additive with ICI-mediated T cell reactivation. Biomarker candidates for identifying patients who benefit from bevacizumab-mediated vascular priming include plasma VEGF-A levels and MRI-based tumor vessel normalization index. This mechanistic rationale supports the investigation of bevacizumab as a combination partner, distinguishing its role in immune modulation from its conventional anti-angiogenic function (120, 121). However, due to the immunosuppressive environment and heterogeneity of GBM, monotherapies have shown limited success (129). To overcome these challenges, combination immunotherapy strategies are being pursued to activate multiple immune pathways simultaneously, enhancing antigen presentation, T cell infiltration, and effector function for more durable responses.

Current and emerging combination immunotherapy approaches can be categorized according to the biological barriers they target. Specific examples include: ICI + ACT (EGFRvIII CAR T + pembrolizumab, NCT03726515: safety confirmed, enhanced CAR T persistence observed); ICI + vaccine (neoantigen peptide vaccine + nivolumab, NCT04717401: ongoing). Regarding OV + vaccine combinations: OV-induced antigen release primes the immune system for neoantigen vaccine strategies, inducing epitope spreading and T cell diversity (87, 130). Other innovative strategies involve remodeling the TME with oncolytic viruses (OVs) to induce tumor lysis or depleting suppressive myeloid cells to enhance CAR T cell persistence. Combining engineered systems that precisely deliver cytokines or cell-based therapies directly to the tumor aims to create a more favorable environment for sustained anti-tumor immunity (131). Radiotherapy (RT) and tumor-treating fields (TTF) are also being investigated in combination with ICIs. RT not only induces cytotoxicity but also promotes immunogenic cell death, increasing antigen availability and enhancing local inflammation to support immune activation (132). TTF (Tumor Treating Fields; 100–300 kHz alternating electric fields) disrupts mitotic spindle assembly via dielectrophoretic forces on tubulin and septin, causing mitotic arrest and immunogenic cell death. Beyond anti-mitotic effects, TTF: (a) increases calreticulin surface expression on tumor cells (a key phagocytic “eat me” DAMP signal); (b) impairs DNA double-strand break repair by disrupting RAD51 foci, potentially activating the STING pathway; (c) upregulates MHC class I on electrically-stressed tumor cells; and (d) promotes DAMP release into the extracellular space. These mechanisms provide mechanistic rationale for TTF + ICI combinations beyond general mitotic disruption (133). Preliminary clinical trials of combination therapies have demonstrated notable results: DCVax-L + standard of care (NCT00045968, Phase III) reported median OS 19.3 months in ITT and 30.2% 3-year OS in a responder subgroup, a potential improvement compared to the 15.6 months MGMT-unselected historical median. RT + nivolumab without TMZ in newly diagnosed MGMT-unmethylated GBM (NCT02617589, CheckMate 498) showed OS 13.4 vs. 14.9 months standard of care—no benefit in unselected population. However, these immune signals (T cell infiltration, antigen-specific responses) have not yet translated to survival improvement in unselected populations, emphasizing the critical need for biomarker-driven patient stratification in future combination trial designs (21, 26, 134).

**Figure 3:**
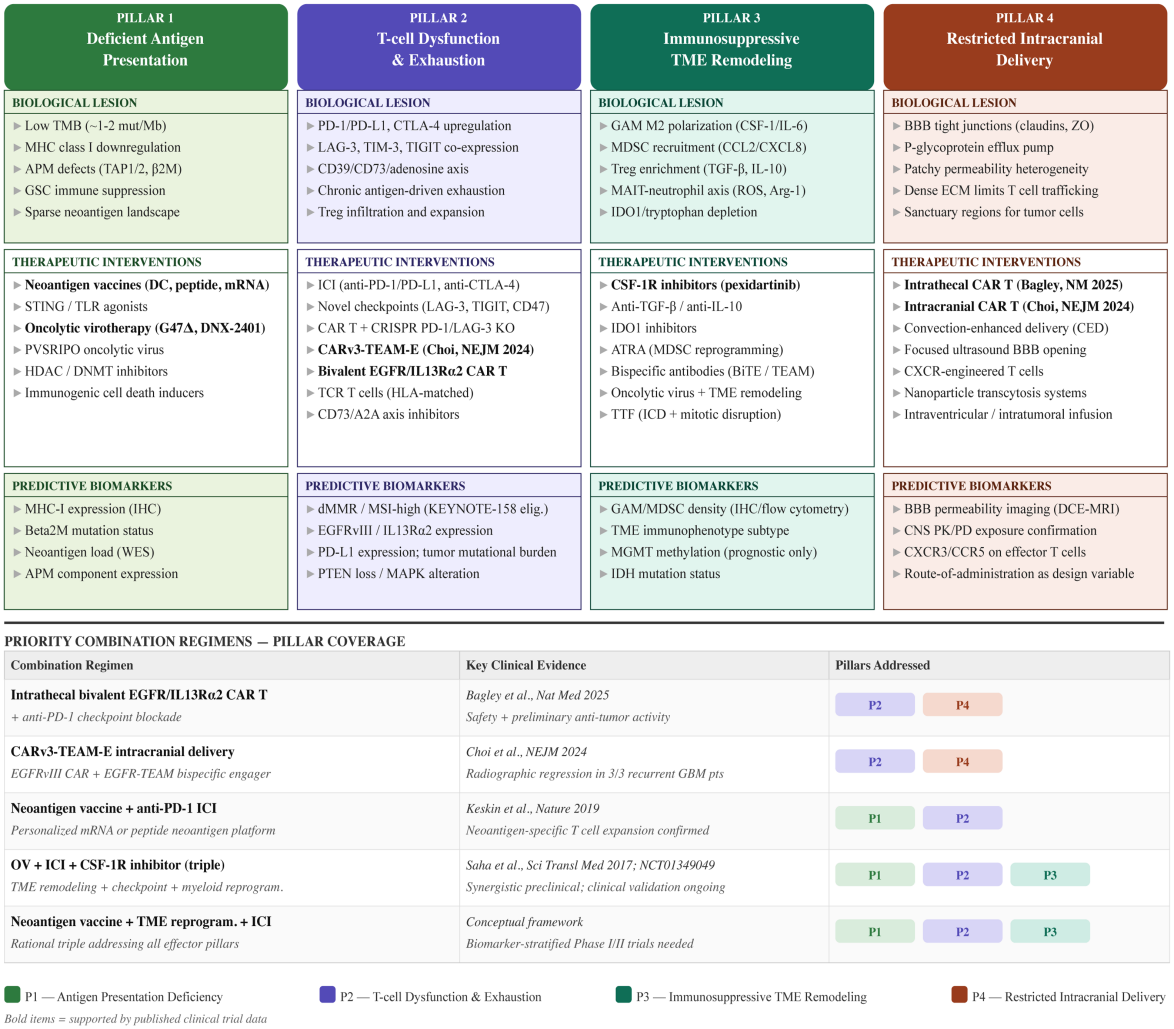
Four core pillars of GBM antitumor immunity: therapeutic strategy mapping and priority combination regimens. Each pillar maps the dominant biological barrier to available interventions, priority combinations, and predictive biomarkers. APM, antigen-processing machinery; BBB, blood–brain barrier; CAR T, chimeric antigen receptor T cell; CED, convection-enhanced delivery; dMMR, mismatch repair deficiency; ECM, extracellular matrix; EGFR, epidermal growth factor receptor; GAM, glioma-associated macrophage/microglia; GSC, glioblastoma stem cell; ICI, immune checkpoint inhibitor; IDH, isocitrate dehydrogenase; IHC, immunohistochemistry; IL13Rα2, interleukin-13 receptor α2; MAIT, mucosal-associated invariant T cell; MDSC, myeloid-derived suppressor cell; MHC, major histocompatibility complex; MSI, microsatellite instability; OV, oncolytic virus; PK/PD, pharmacokinetics/pharmacodynamics; TAP, transporter associated with antigen processing; TEAM, T cell-engaging antibody molecule; TMB, tumor mutational burden; TME, tumor microenvironment; Treg, regulatory T cell; TTFields, tumor treating fields; WES, whole-exome sequencing; ZO, zonula occludens.

Despite these advances, combination strategies face challenges like toxicity, immune-related adverse events, and complex dosing and scheduling. Multi-agent therapies also present regulatory and manufacturing difficulties. Nevertheless, personalized, rationally designed combinations that integrate ICIs, vaccines, ACT, OVs, and adjunct therapies such as RT and TTF offer the most promising route for improving GBM treatment outcomes. To provide a holistic view of how these complex interactions and clinical efforts are structured, we have visually synthesized these multifaceted strategies in Figure 3 (see Section 5.3), which maps specific interventions onto the core biological pillars of GBM immunity.

## 5 Persistent challenges and innovative responses

GBM remains a moving target: tumor-specific antigens are scarce and uneven, so canonical targets (EGFRvIII, IL13Rα2, and HER2) are patchy or absent across patients, enabling immune escape (135). GSCs add low-immunogenic, distinct antigenic programs that drive plasticity and evasion (136). The TME layers suppression (TGF-β, IL-10, metabolic brakes), while systemic lymphopenia and impaired dendritic cells blunt priming—together arguing for strategies that enhance antigen presentation, reprogram the TME, and improve trafficking/persistence (137).

### 5.1 Overcoming antigen heterogeneity and CAR T cell exhaustion

The antigenic heterogeneity of GBM presents a major barrier to the efficacy of single-target immunotherapies. Advances in single-cell sequencing have enabled the identification of clonal neoantigens, facilitating more precise target development for immunotherapies. To address tumor plasticity and reduce the risk of immune escape, next-generation CAR T cells are being engineered with multi-target (dual- or tri-specific) designs, enabling simultaneous recognition of multiple glioma-associated antigens. Critically, recent clinical evidence has validated this approach: Choi et al. demonstrated that the CARv3-TEAM-E construct—combining an EGFRvIII-directed CAR with a secreted EGFR-targeting T-cell engager—produced rapid radiographic responses in recurrent GBM patients via intracranial delivery, overcoming the antigen-loss escape that limits single-target EGFRvIII CAR T cells (125). Complementarily, Bagley et al. reported that a bivalent EGFR/IL13Rα2 CAR T, delivered intrathecally, simultaneously addressed antigen heterogeneity and the BBB delivery barrier, providing translational proof-of-concept for multi-antigen, locally administered CAR T strategies (126, 138). Concurrently, genome editing technologies like CRISPR/Cas9 are being used to combat CAR T cell exhaustion by deleting inhibitory receptors such as PD-1, LAG-3, and TIM-3. These innovations aim to enhance CAR T cell persistence and cytokine production, thereby promoting sustained anti-tumor activity within the challenging GBM TME. Personalized therapies, including TCR T cells and neoantigen-based vaccines, are advancing rapidly, driven by AI-powered epitope prediction tools. Current tools include pVACtools (integrating NetMHCpan, NetMHCIIpan, and other predictors to prioritize MHC class I/II epitopes from somatic mutation data), MHCflurry (a deep learning-based pan-MHC predictor), and the TESLA consortium benchmark demonstrating that ensemble prediction approaches outperform single-tool methods. Key limitations include: poor MHC class II prediction accuracy compared to class I; failure to account for antigen processing efficiency (TAP transport, proteasomal cleavage); and limited training data for HLA alleles outside European populations. Neoantigen clonality—the fraction of tumor cells harboring a given mutation—is now recognized as a key target selection criterion: clonal neoantigens present in all tumor cells are preferred over subclonal ones as they minimize the risk of immune escape; single-cell sequencing approaches are increasingly used to estimate clonality in prospective vaccine trials such as NCT04280848.

### 5.2 Engineering delivery systems to overcome the blood–brain barrier

A critical obstacle in GBM immunotherapy is the restricted access of therapeutic agents across the BBB. Innovative delivery systems are being developed to address this challenge, including the engineering of CAR T cells to secrete antibody fragments that improve intratumoral trafficking and convection-enhanced delivery (CED) to bypass the BBB (53) and the engineering of CAR T cells to secrete antibody fragments that enhance intratumoral trafficking. Additionally, nanoparticle encapsulation of cytokines or immune modulators, along with intracranial infusion systems, is being investigated to localize immune activity within the tumor bed while minimizing systemic toxicity. Notably, the intrathecal delivery strategy employed in the bivalent EGFR/IL13Rα2 CAR T trial (126) exemplifies a clinically actionable BBB-bypass approach. By achieving sustained intracranial CAR T cell presence and anti-tumor activity without systemic infusion, this trial strengthens the case for route-of-administration as a pivotal design variable in future GBM trials. Furthermore, advances in neoantigen prediction, supported by next-generation sequencing and artificial intelligence (AI), are refining the precision of vaccines and TCR-based therapies (138). These technological innovations, coupled with next-generation models such as patient-derived organoids (PDOs) and humanized mouse systems, allow for a more faithful replication of human GBM immunobiology and more predictive preclinical testing. Ultimately, integrating optimized drug delivery with personalized immune-oncology aims to enhance therapeutic specificity, immune cell infiltration, and persistence for more durable clinical outcomes.

### 5.3 Synthesis: mapping therapeutic strategies onto the four core pillars of GBM immunity

The preceding modality-by-modality review converges on a unifying framework: durable immune control of GBM will require concurrent addressing of four core biological pillars—(1) deficient antigen presentation, (2) T-cell dysfunction and exhaustion, (3) immunosuppressive TME remodeling, and (4) restricted intracranial delivery. Below we map the available and emerging interventions onto each pillar and identify priority combination regimens supported by the current evidence base (Figure 3).

*Pillar 1*—Deficient antigen presentation: GBM’s low tumor mutational burden, MHC class I downregulation, and impaired antigen-processing machinery (TAP1/2, β2M, immunoproteasome subunits) limit CD8⁺ T cell priming (see Sections 2.1 and 2.4). The interventions most directly targeting this pillar are: (a) personalized neoantigen tumor vaccines (122, 123); (b) STING/TLR agonists and oncolytic virotherapy [G47Δ (104); DNX-2401 (139); PVSRIPO (140)], which trigger immunogenic cell death and activate dendritic cell cross-presentation via DAMPs/PAMPs; and (c) epigenetic modulators (DNMT/HDAC inhibitors) that de-repress silenced tumor antigens. Relevant predictive biomarkers for this pillar: MHC class I expression by IHC, β2M mutation status, and neoantigen load by whole-exome sequencing.

*Pillar 2*—T-cell dysfunction and exhaustion: Chronic antigen exposure, checkpoint upregulation (PD-1/PD-L1, CTLA-4, LAG-3, TIM-3, TIGIT), and the adenosinergic CD39/CD73 axis cooperatively drive T cell exhaustion in GBM (21, 29). ICIs directly target this pillar, though single-agent efficacy has been limited (CheckMate 143, NCT02017717; CheckMate 498, NCT02617589); combination with radiotherapy and neoantigen vaccines has shown additive immunological activity in early trials. CAR T and TCR T cells engineered with CRISPR/Cas9 deletion of PD-1 or LAG-3 represent next-generation products that intrinsically resist exhaustion (125, 126). It is essential to distinguish, predictive biomarkers (e.g., EGFRvIII expression for CAR T; PD-L1 expression and TMB for ICI) from prognostic biomarkers (e.g., MGMT methylation), to refine patient selection and avoid confounding trial outcomes.

*Pillar 3*—Immunosuppressive TME remodeling: GAMs, MDSCs, and Tregs form a mutually reinforcing immunosuppressive network (see Section 2.2). Targeting this pillar requires strategies that shift the myeloid balance [CSF-1R inhibitors, e.g., pexidartinib (NCT01349049); ATRA for MDSCs], disrupt suppressive cytokine gradients (anti-TGF-*β*, anti-IL-10), or metabolically reprogram the TME (IDO1 inhibitors, CD73/A_2A_-axis blockade). CART. BiTE constructs and CAR-TEAM systems (125) represent emerging engineered approaches that combine direct tumor killing with local TME immune activation. Priority combinations: OV + ICI (127); CAR T + anti-PD-1; and neoantigen vaccine + TME reprogramming + ICI (triple combination addressing Pillars 1 + 2 + 3).

*Pillar 4*—Restricted intracranial delivery: The BBB remains the fundamental pharmacokinetic barrier for both small molecules and cellular therapies (see Section 2.3). Delivery-focused interventions range from physical approaches, such as focused ultrasound (FUS)-mediated BBB opening (59, 141), CED, and intraventricular infusion, to biological strategies like CXCR engineering of T cells. Notably, the clinical validation of intrathecal bivalent EGFR/IL13Rα2 CAR T delivery (126) provides compelling translational evidence that route-of-administration engineering can overcome the BBB, achieving intracranial CAR T concentrations sufficient for anti-tumor activity without systemic toxicity. Future GBM trial designs should pre-specify delivery route and pharmacokinetic/pharmacodynamic endpoints to confirm intracranial target engagement.

In summary, no single therapeutic modality is sufficient to overcome GBM’s multidimensional immune resistance. The most therapeutically compelling combinations are those that simultaneously address at least two pillars: intrathecal bivalent CAR T (Pillars 2 + 4) (126), neoantigen vaccine + ICI (Pillars 1 + 2; NCT04417764), OV + ICI (Pillars 1 + 2) (142), and OV + ICI + myeloid CSF-1R blockade (Pillars 1 + 2 + 3). Prospective biomarker-stratified trials with pre-specified pillar-relevant endpoints—incorporating tumor neoantigen load, TME immunophenotype, and BBB permeability assessments—will be essential to validate these mechanistically informed combination hypotheses.

## 6 Conclusion and future directions

Although the overall clinical efficacy of immunotherapy in GBM remains limited to date, the field is undergoing rapid transformation driven by sustained innovation in targeting strategies, expanding combination regimens, and the advancing frontiers of personalized medicine. Over the past decade, the development of novel immune ICIs, engineered cellular therapies, and tumor vaccines tailored to individual antigenic profiles has demonstrated that even in a profoundly immunosuppressive and heterogeneous tumor like GBM, carefully designed immunotherapeutic approaches can elicit meaningful biological responses. Looking forward, technological innovation is poised to play a central role in overcoming current therapeutic bottlenecks. Stem cell–derived immune effector cells, such as iPSC-based CAR T or CAR NK products, offer virtually unlimited expansion potential, enabling the production of standardized, off-the-shelf therapies with enhanced scalability and persistence. At the same time, a next-generation engineering paradigm is emerging, integrating AI-based neoantigen prediction, CRISPR/Cas9-mediated rapid construction of personalized CAR designs, and patient-derived organoid models for preclinical functional screening—collectively streamlining the development of precise, individualized cellular therapies. These advancements, coupled with efforts to extend immunotherapy into earlier disease stages such as lower-grade gliomas or postoperative recurrent GBM, may open new therapeutic windows and improve long-term outcomes. Ultimately, meaningful progress will require not only technical innovation but also robust interdisciplinary collaboration across immunology, oncology, biomedical engineering, and translational science to design high-quality, biomarker-guided clinical trials. With sustained investment and integration of these diverse fields, the prospect of delivering durable, patient-specific immunotherapeutic benefit to glioma patients is becoming increasingly tangible.
